# Influence of Multiple Cardiovascular Risk Factors on Task-Switching in Older Adults: An fMRI Study

**DOI:** 10.3389/fnhum.2020.561877

**Published:** 2020-09-09

**Authors:** Shuo Qin, Chandramallika Basak

**Affiliations:** Center for Vital Longevity, The University of Texas at Dallas, Dallas, TX, United States

**Keywords:** cardiovascular risk factors, aging, task-switching, fMRI, age-related difference

## Abstract

Not only are the effects of cardiovascular risk factors such as high blood pressure and low fitness on executive functions and brain activations in older adults scarcely investigated, no fMRI study has investigated the combined effects of multiple risk factors on brain activations in older adults. This fMRI study examined the independent and combined effects of two cardiovascular risk factors, arterial plasticity, and physical fitness, on brain activations during task-switching in older adults. The effects of these two risk factors on age-related differences in activation between older and younger adults were also examined. Independently, low physical fitness and low arterial plasticity were related to reduced suppressions of occipital brain regions. The combined effects of these two risks on occipital regions were greater than the independent effects of either risk factor. Age-related overactivations in frontal cortex were observed in low fitness older adults. Brain-behavior correlation indicates that these frontal overactivations are maladaptive to older adults’ task performance. It is possible that the resulting effects of cardiovascular risks on the aging brain, especially the maladaptive overactivations of frontal brain regions by high risk older adults, contribute to often found posterior-anterior shift in aging (PASA) brain activations. Furthermore, observed age-related differences in brain activations during task-switching can be partially attributed to individual differences in cardiovascular risks among older adults.

## Introduction

The normal aging process is accompanied by increased cardiovascular burden such as hardened arterial walls and high blood pressure (Safar et al., 2012; Homan and Cichowski, 2019). Most medical professionals suggest a physically active lifestyle as the most cost-effective method to reduce the negative effects of cardiovascular burden (Carpio-Rivera et al., 2016). Still, increased sedentary behavior due to physical frailty is common among the older population (Diaz et al., 2016). Behavioral studies showed that increases in these cardiovascular risks (i.e., cardiovascular burden and sedentariness) are associated with worse performance in cognitive functions already impacted by the aging process, such as executive functions (exaggerated age-related differences; Colcombe and Kramer, 2003; Gifford et al., 2013; Prakash et al., 2015; Rodrigue and Bischof, 2016). The extent to which increased cardiovascular risks exaggerate observed age-related differences in brain functions between older and younger adults has never been directly examined. Although neuroimaging studies have reported exaggerate declines in brain volumes associated with high blood pressure or high sedentariness in older adults (Raz et al., 2005; Erickson et al., 2009), the effects of such cardiovascular risk factors on brain activations during task, especially during tasks of executive functions, have not been investigated.

Two cardiovascular risk factors that are highly prevalent in older adults are decreased arterial plasticity, indicated by increases in blood pressure and pulse pressure (Dart and Kingwell, 2001; Weiss et al., 2009), and decreased physical fitness, indicated by decrease in VO_2_Max (Hagberg, 1987; Fitzgerald et al., 1997). Behaviorally, both decreased arterial plasticity and decreased fitness have been associated with worse executive functions in older adults (Nishtala et al., 2014; for a review, see Bherer et al., 2013). In terms of brain functions, both increase in measure of arterial plasticity (e.g., blood pressure) and decrease in measure of physical fitness (e.g., VO_2_Max) have been associated with reduced overall cardiovascular reactivity (Haight et al., 2015; Tsvetanov et al., 2019) and reduced cerebral blood flow (Ainslie et al., 2008; Tarumi and Zhang, 2018). Such reduction in cardiovascular reactivity and cerebral blood flow may in turn influence blood-oxygen-level-dependent (BOLD) signals from functional magnetic resonance imaging (fMRI) studies. The effects of these two risk factors, arterial plasticity and physical fitness, on fMRI brain activations (that is, the BOLD signals), after controlling for their effects on cardiovascular reactivity and cerebral blood flow, in older adults are not clearly understood. To our knowledge only one neuroimaging study has reported increases in fMRI brain activations (in the inferior parietal lobule) to be associated with high blood pressure in older adults during a task of executive functions (Flanker task; Chuang et al., 2014). There has been no study till date that has examined the effects of physical fitness on whole-brain activations in older adults using tasks of executive functions. Most importantly, no study has ever investigated the combined effects of arterial plasticity and physical fitness on brain activations during executive function tasks in older adults.

Most non-clinical studies that examined “healthy” older adults recorded participants’ intake of blood pressure medications or their amounts of daily exercise based on self-report. As a result, for any given sample of “healthy” older adults, it is highly possible to contain some older adults with high blood pressure controlled by either medication or physical exercise, or both. The effects of one factor (e.g., arterial plasticity) on executive functions and brain activations in older adults therefore cannot be completely independent of the effects of the other factor (e.g., physical fitness). Rather, it would be feasible to study the combined effects of multiple factors on older adults’ executive functions and its related brain activations. How do sedentary older adults with hardened arterial wall differ from active older adults with high arterial plasticity? The current study aimed to investigate not only the separate, but also the combined effects of the two cardiovascular risk factors on older adults’ brain activations.

The current study focuses on one key executive functions subprocess – task-switching. The ability to switch attention from one task to another, an important subprocess of executive functions (Miyake et al., 2000), plays a critical role in daily functioning. Older adults who complain about difficulties in driving or multitasking are often having problems in cognitive abilities measured by task-switching paradigms (McAlister and Schmitter-Edgecombe, 2016). Typical task-switching paradigms consist of two types of task blocks: (1) Single task blocks where participants have to perform a single task per block, and (2) Dual task blocks where participants have to perform the two tasks in the same block such that switching between the tasks is necessitated by a visual cue (e.g., a change of background color or stimuli color). These paradigms therefore yield metrics of two main types of cognitive control: a global switch cost (GSC) and a local switch cost (LSC). GSC is the reaction time (or brain activation) difference between single and dual task blocks. LSC is the reaction time (or brain activation) difference between switch trails, where task changes from previous trial, and non-switch trials, where the task remains unchanged, within Dual task blocks (Braver et al., 2003; Jimura and Braver, 2010; Kim et al., 2012; Eich et al., 2016; Nashiro et al., 2018). Neuroimaging studies on task-switching paradigms have consistently found fronto-parietal brain activations in younger adults for GSC and LSC (for meta-analyses see, Wager et al., 2004; Kim et al., 2012). In line with these results, a recent study from our lab that used the same task-switching paradigm as the current study reported five fronto-parietal brain regions that showed significant GSC activations, and a right post-central gyrus regions that showed significant LSC activation (Nashiro et al., 2018).

Age-related differences in brain activations during task-switching paradigms have been reported by multiple neuroimaging studies (Jimura and Braver, 2010; Steffener et al., 2014; Hakun et al., 2015; Berry et al., 2016; Eich et al., 2016; Nashiro et al., 2018). Older adults typically show increased activations in regions not activated by younger adults, especially in frontal and parietal brain regions. Such additionally activated regions in older adults are termed overactivations (Grady et al., 1994). For task-switching paradigms, overactivations have been associated with worse performance in older adults, suggesting the need to activate additional regions in attempt to compensate for tanking performance (Prakash et al., 2012; Eich et al., 2016; Nashiro et al., 2018). However, the effects of individual differences, such as cardiovascular risks, among older participants on these maladaptive overactivations have not been examined.

The current fMRI study aimed to investigate the effects of arterial plasticity and physical fitness, separately and in combination, on (1) the whole brain activations in older adults, and (2) task-sensitive activations in *a priori* regions of interests (ROIs) defined from a previous publication (Nashiro et al., 2018). The *a priori* ROIs were defined from an independent sample of adults through whole-brain contrasts that examined the brain activations associated with GSC and LSC during the same task-switching paradigm as used in the current study. Brain activations from these six task-sensitive ROIs (five ROIs for GSC and one ROI for LSC) did not differ between younger and older adults (Basak et al., 2018; Nashiro et al., 2018). We hypothesize that increase in one cardiovascular risk factor (i.e., low arterial plasticity or physical fitness) will not only be associated with worse performance during this executive functions task, but will also be associated with distinct patterns of brain activations. We predict that individuals with one or more risk factors will show either (1) increased overactivations in brains regions beyond the six task-sensitive ROIs, suggesting an attempt by the at-risk brain to compensate for the tanking performance, or (2) decrease in activation in the six task-sensitive ROIs, suggesting a failure to sufficiently engage task-sensitive regions by at-risk older adults when compared to younger adults and healthier older adults, or (3) both attempted compensation and decreased engagement of task-sensitive regions. We further hypothesize that these patterns of brain activations in older adults will be greatly exaggerated with the combined effects of the two risk factors, when compared to a single factor alone, thus implicating the additive effects of these risk factors on the aging brain.

## Materials and Methods

### Participants Characteristics

A total of 60 older adults (*M_age_* = 67.72, *SD_age_* = 6.87; *M*_*MoCA*_ = 28.13, *SD*_*MoCA*_ = 1.6) were recruited from the Dallas Fort-Worth metroplex. All participants were screened for medical, neurological, or psychiatric illnesses. Other exclusion criteria included current or previous substance abuse, less than a high school education, diabetes, left-handedness, depression (assessed by the Geriatric Depression Scale; Burke et al., 1991), psychiatric disorders, color blindness (assessed by the Ishihara Color Blindness test), and a Montreal Cognitive Assessment (MoCA) score of less than 24. All participants underwent screening in a mock scanner to ensure their MRI compatibility. Participants’ blood pressure and resting heart rate were taken twice during the hour-long screening session, once at the beginning and once toward the end. The second readings of blood pressure and resting heart rate were used in analyses. Heights and weights of participants were measured by standard scales available at the University of Texas Southwestern Medical Center. Participants signed an informed consent, approved both by the University of Texas at Dallas and the University of Texas Southwestern Medical Center Institutional Review Boards and were paid $40 for the MRI session and $15 for the screening session.

In addition, brain activations from a sample of younger adults (*N* = 28, *M_age_* = 25, *SD_age_* = 3.16) published by Nashiro et al. (2018) were included as a functional control group. The younger adults were healthy and active undergraduate and graduate students attending classes at the University of Texas at Dallas. Most younger adults have body mass index (BMI) in the lean to fit range (*M_BMI_* = 21.9, *Range* = 16–25), with one participant’s BMI falling in the “overweight” category (BMI = 26; Weir and Jan, 2019). No whole-brain analysis was performed on this younger sample.

### Assessment of Arterial Plasticity and Physical Fitness

Arterial plasticity was assessed via pulse pressure (Thorin-Trescases et al., 2018). Pulse pressure (*PsP*) for each participant was calculated via subtracting diastolic blood pressure from systolic blood pressure taken during the screening session. High *PsP* indicates low arterial plasticity. *PsP* is a direct measure of arterial stiffness, especially in older adults whose blood vessels tend to “harden” (i.e., reduced plasticity) due to reasons such as calcification (Blacher et al., 2000; Safar et al., 2012; Homan and Cichowski, 2019).

The “gold standard” measure for physical fitness is VO2Max, which is a measure of the maximum oxygen consumption in an individual’s body obtained during a physical exhaustion test. However, older and low-fit individuals may have conditions that prevent them from participating in the physical exhaustion test. In the current study, the metabolic equivalent (MET) of VO2Max was estimated based on the following equation (Jurca et al., 2005):

(1) MET=Gender(male=1,female=0)×2.77-age×0.1+P⁢h⁢y⁢s⁢i⁢c⁢a⁢l⁢a⁢c⁢t⁢i⁢v⁢i⁢t⁢y⁢s⁢c⁢a⁢l⁢e+18.07+P⁢h⁢y⁢s⁢i⁢c⁢a⁢l⁢a⁢c⁢t⁢i⁢v⁢i⁢t⁢y⁢s⁢c⁢a⁢l⁢e+18.07

This measure utilizes easily acquired parameters that are highly predictive of VO_2_Max (Jurca et al., 2005). It has been demonstrated to approximate VO_2_Max with good accuracy in a large sample (N > 10,000), and recently, Mailey et al. (2010) extended the validity of the MET estimate specifically to older adults, ranging in ages from 60 to 80. McAuley et al. (2011) further validated this measure for smaller sample sizes by showing that there was no significant difference between this estimated measure of MET and the gold-standard, physician-supervised, maximal exercise test in a sample of 86 older adults. Finally, in a survey of over 32,000 individuals ranging in age from 35 to 70 years, Stamatakis et al. (2013) found MET to be a good predictor of cardiovascular (and overall) mortality, comparable to associations between exercise testing cardiovascular fitness and mortality. High MET indicates high physical fitness. Table 1 reports the statistics for *PsP*, MET, and main demographic variables for older participants. Participants were classified in the low arterial plasticity group (*N* = 17) if having a *PsP* of 60 and above (Weiss et al., 2009), and in the high arterial plasticity group (*N* = 43) if having a *PsP* of 59 and below. Based on published normative data, healthy older adults should have a MET score over 5 (Jurca et al., 2005). For the current sample of older adults, MET ranges between 2.9 and 12.53, with most participants having METs over 5 (*N* = 49). Participants were characterized into the high fitness and low fitness groups based on a median split of MET = 7.4. In order to investigate the combined effects of arterial plasticity and physical fitness on brain, older adults were further separated into three groups: those with no risk factor (high arterial plasticity and fitness; *N* = 25), those with one risk factor (low arterial plasticity or low fitness; *N* = 24), and those with two risk factors (low arterial plasticity and low fitness; *N* = 11).

**TABLE 1:** Characteristics of older participants, first separated into high and low arterial plasticity groups based on *PsP*, then separated into high and low physical fitness groups based on MET.

	**Low Plasticity**	**High Plasticity**
*N* (M/F)	17 (7/10)	43 (15/28)
Age (Range)	70.65 (60–81)	66.74 (60–86)
Sys. BP (Range)	145.76 (125–168)	124.72 (94–142)
Dia. BP (Range)	76.59 (62–86)	81.09 (60–106)
BMI (Range)	25.71 (19–37)	27.36 (18–30)
*PsP* (Range)	69.18 (61–97)	43.63 (24–59)
MET (Range)	6.83 (4.37–11.08)	7.53 (2.65–12.53)
MoCA (*SD*)	28.45 (1.43)	27.53 (1.84)

	**Low Fit**	**High Fit**

*N* (M/F)	30 (5/25)	30 (19/11)
Age (Range)	67.23 (60–86)	68.53 (60–81)
Sys. BP (Range)	131.20 (105–159)	130.33 (94–168)
Dia. BP (Range)	78.93 (62–95)	80.63 (60–106)
BMI (Range)	28.04 (18–37)	24.25 (19–30)
*PsP* (Range)	52.27 (27–78)	49.7 (24–97)
MET (Range)	5.32 (2.62–7.36)	9.16 (7.54–12.53)
MoCA (SD)	27.94 (1.65)	28.39 (1.57)

*N, sample size; Sys. BP, systolic blood pressure; Dia. BP, diastolic blood pressure; BMI, body mass index; *PsP*, pulse pressure; MET, metabolic equivalent of VO_2_Max; MoCA, Montreal Cognitive Assessment score.*

Like VO2Max (Bouchard et al., 1998), the MET score is biased toward male participants. Age has traditionally been associated with cardiovascular conditions in older adults. Therefore, gender and age were controlled for in all analyses.

### Analysis of Statistical Power

Our two key analyses are whole-brain contrasts between the following groups of older adults: (1) high fitness vs. low fitness, and (2) high plasticity vs. low plasticity. We, therefore, computed power analysis assuming a two sample *t*-test for each of these two key analyses. We selected an effect size based on a past neuroimaging study that had examined brain activation differences between high and low fit older adults during an flanker task (Colcombe et al., 2004). Given the dearth of neuroimaging studies on executive functions that have explored fitness differences in brain activations in a normal aging sample, who also have moderate fitness levels, we selected this study as it is the closest to our sample and our analysis approach, although it used a different executive function process. In the Colcombe et al. (2004) study, brain regions with significant differences in activations between high (*N* = 20) and low (*N* = 21) fit older adults had unit-normal *Z* scores (converted from *t*-statistic) ranging between 2.58 and 4.49, corresponding to the Cohen’s *d* effect sizes of 0.82 to 1.44. We selected a conservative estimate, that is the lowest effect size (*d* = 0.82), for our power calculations with a Type I error rate of 0.05 (α = 0.05) using G^∗^Power 3.1 package (Faul et al., 2007).

With the analysis described above, a sample size of *N* = 60 subjects (*n* = 30 for each group) guarantees a power of 93% to detect a significant difference between the high fitness and low fitness groups. For the plasticity contrast, our sample size of *N* = 60 [*n* = 43 (high plasticity); *n* = 17 (low plasticity)] provides a power of 88% to detect a significant difference between the groups. In addition to these primary analyses, we also conducted an additional analysis to evaluate the combined effects of cardiovascular risks (0 risk factor vs.1 risk factor vs. 2 risk factors). With this analysis, a sample size of *N* = 60 subjects guarantees a power of 80% to detect a main effect of group.

### Task-Switching Paradigm

**FIGURE 1:**
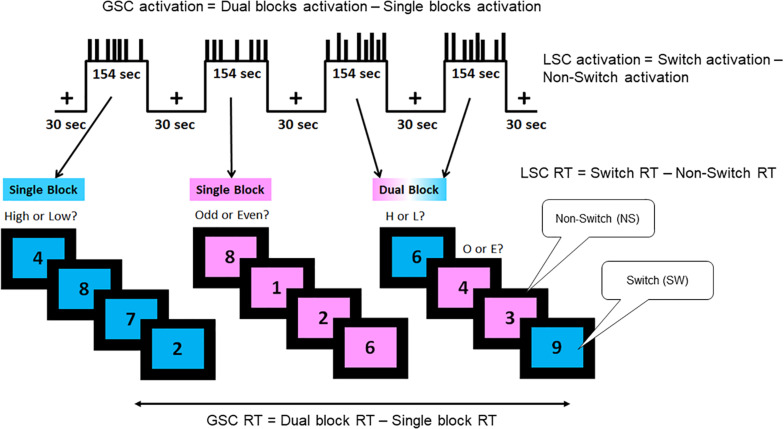
Task-switching paradigm. A hybrid block/event-related design was used, which consisted of two single blocks and two dual blocks interspersed by fixation periods. The short bars in the task blocks represent the non-switch trials, and the long bars represent the switch trials. The blue background required judgment of whether the digit shown was lower or higher than five, while the pink background required judgment of whether the digit shown was odd or even. There were three trial types as follows: single, dual non-switch, and dual switch. Two types of cognitive control processes were assessed by this paradigm: global switch cost (GSC) and local switch cost (LSC). Behaviorally, GSC was indicated by the reaction time differences between single task blocks and dual task blocks. LSC was indicated by the reaction time differences between switch and non-switch trials. In neuroimaging analyses, GSC was assessed by the brain activation differences between single and dual blocks (block-design analysis). LSC was assessed by the brain activation differences between switch and non-switch trials within dual blocks (event-related analysis). Adapted from Nashiro et al., 2018.

Description of the task is adapted from previous publications (Basak et al., 2018; Nashiro et al., 2018; Figure 1). A single digit (1–9, excluding 5) was presented in the center of the screen, on either a pink or blue background (Figure 1). There were two different tasks, indicated by the background color of the digit: (1) judging whether a number was lower or higher than five if the background was blue, and (2) judging whether a number was odd or even if the background was pink. Participants were instructed to press the left button for lower/odd and the right button for higher/even judgments. There were a total of 120 trials separated equally into four task blocks with each task block lasting 154 s. The first two blocks were single blocks (one block of low/high task and one block of odd/even task), followed by two dual blocks, in the task for each trial was selected randomly. Half of the trials in the dual-task blocks were switch trials in which the task from the preceding trial was different than that of the current trial (e.g., low/high to odd/even); the other half were non-switch trials where the same task was repeated across two consecutive trials (e.g., low/high to low/high).

The paradigm measures two types of executive control functions, GSC and LSC. GSC is calculated by the reaction time difference between single task blocks and dual task blocks. LSC is calculated by the reaction time difference between switch trials and non-switch trials within the dual blocks. In fMRI analysis, GSC activation is captured by activation differences between single and dual blocks via block design analysis. LSC activation is captured by activation differences between switch and non-switch trials within dual blocks via event-related design analysis.

### Scanning Procedures

Scanning was performed with a Philips Achieva 3T MR scanner (Philips Medical Systems, Andover, MA, United States). High-resolution anatomical images were acquired, using a transverse MPRAGE T1-weighted sequence with the following parameters (TR = 8.1 ms; TE = 3.7 ms; flip angle = 12°; acquisition matrix = 256 × 204; voxel size = 1 mm^3^; 160 slices).

Functional images were acquired using an echo-planar sequence (TR = 2000 ms; TE = 30 ms; flip angle = 70°; acquisition matrix = 64 × 64; voxel size = 3.44 × 3.44 × 4 mm; 39 axial slices). We used a hybrid blocked and event-related design (e.g., Braver et al., 2003; for a review, see Petersen and Dubis, 2012). The task consisted of alternating cycles of the task (T) and fixation (F) blocks with the following structure: F, T, F, T, F, T, F, T, F. Each fixation block was of 30 s duration, and the four task blocks were of 154 s duration each. Each task block had 30 trials in which a stimulus was presented for 3 s, within which the participant responded, followed by a fixation cross. To optimize stimulus sequence and timing the inter-trial interval (ITI) ranged from 1.5 to 5 s with a mean ITI of 2.13 s.

### Imaging Data Analyses

#### Preprocessing

The first seven EPI volumes were dropped to allow the signal to reach steady-state magnetization. FSL 6.0 was used for fMRI data preprocessing. MELODIC (Multivariate Exploratory Linear Optimized Decomposition into Independent Components) preprocessing and FIX (FMRIB’s ICA-based Xnoiseifier) denoising were performed on individual fMRI data to remove physiological artifacts such as motion, respiration, and cerebrovascular conditions from blood oxygenation level-dependent (BOLD) signals. Within MELODIC preprocessing, high-pass temporal filtering was set to 380 s for block design analysis and 100 s for event-related design analysis. Linear registration was performed between the functional and structural images using affine boundary-based registration (Greve and Fischl, 2009). The structural images were normalized to the study-specific template, created from all included older participants, by linear registration (FLIRT tools from FSL; Jenkinson and Smith, 2001; Jenkinson et al., 2002). After MELODIC preprocessing, resulting independent components were manually classified as “noise” and “signal” based on location of significant clusters, power spectrum and time series of each component (Kelly et al., 2010). After manual classification, components identified as 100% noise-related were removed from preprocessed data using the FIX. Resulting data cleaned of physiological artifacts were spatially smoothed with a 4 mm full-width at half-maximum (FWHM) Gaussian kernel.

#### General Linear Modeling (GLM). Whole-Brain GLM Fittings Were Conducted Using FSL FEAT 6.00

##### Block design analyses

For each run in every participant, stimulus-dependent changes in BOLD signal were modeled with two regressors (i.e., single and dual blocks). The fixation blocks were modeled as the baseline level of activity and therefore, were not included as a regressor. The regressors were convolved with a gamma hemodynamic response function, including the six head movement parameters as confounds. Temporal filtering was applied. For the first-individual level analyses, the amplitude of the hemodynamic response was estimated to calculate GSC (dual blocks > single blocks). The resulting images were then entered into the group analyses to obtain an average across all participants.

##### Event-related analyses

For each run in every participant, stimulus-dependent changes in BOLD signal were modeled with four regressors: (1) single, (2) non-switch, (3) switch, and (4) error trials. The regressors were convolved with a gamma hemodynamic response function, including the six head movement parameters as confounds. Temporal filtering was applied, and temporal derivatives of each of the regressors were also included. LSC regions were determined by the difference between switch trials (SW) and non-switch trials (NS) and were represented by the SW > NS contrast. The resulting images were then entered into the group analyses to obtain an average across all participants.

### fMRI Group Analyses

To control for confounding effects of cardiovascular conditions on BOLD signals, a residual variance value was calculated for each participant as a proxy for BOLD signal variability, which has been suggested by recent studies as associated with systematic differences in cerebral vascular reactivity in older adults (Liu et al., 2013; Tsvetanov et al., 2015). For each participant, a residual variance image was created from GLM fitting detailed in section “General Linear Modeling (GLM). Whole-Brain GLM Fittings Were Conducted Using FSL FEAT 6.00.” In addition, volume parcelation (FAST, Zhang et al., 2001) was conducted for each participant to create partial gray matter volume masks. The residual variance value was calculated by first registering the residual variance image onto partial gray matter masks for each participant. Then the residual variance values from each voxel were averaged across space to acquire a single estimate of BOLD signal variability within gray matter, which was entered as an individual regressor in group analyses. In addition, individual age and gender were included as covariates in group analyses.

Whole-brain contrasts were conducted between older adults in (1) high arterial plasticity and low arterial plasticity groups, (2) high fitness and low fitness groups, and (3) 0 risk factor (high arterial plasticity and high fitness) and 2 risk factors (low arterial plasticity and low fitness). Analyses were conducted for both GSC (block design) and LSC (event-related design). Z (Gaussianised T/F) statistic images were thresholded at the whole-brain level using the clusters thresholding option in FEAT at *z* > 2.58. The voxels that passed the threshold were combined into clusters. Random field theory (RFT) was then used to give the *p*-value of obtaining a cluster of voxels given the set spatial smoothness and the *z* threshold used. These *p*-values were thresholded at a *p* = 0.01 to obtain “significant” clusters.

Mean% signal changes from significant clusters identified by whole-brain group contrasts were extracted from both older adults and younger adults and plotted for visual inspection of activation patterns. Independent sample *T*-test between mean% signal changes from younger adults and mean% signal changes from older adults groups (high and low arterial plasticity; high and low fitness) were conducted to assess age-related differences in arterial plasticity-sensitive and fitness-sensitive clusters.

For clusters defined as showing combined effects of the two risk factors (0 factors vs. two factors), mean% signal changes from the 1-risk factor group (high risk or low fitness alone) were also extracted and compared to both 0- and 2-risk factors groups to assess if combined effects of low arterial plasticity and low physical fitness on brain activations were greater than separate effect of arterial plasticity or fitness alone on brain activations.

Brain-behavior prediction analyses were conducted in older adults to examine whether activations from these regions were predictive of task performance. GSC RT (dual RT- single RT) and LSC RT (switch RT – non-switch RT) were included as dependent variables in brain-behavior prediction analyses. Only significant brain-behavior relationships were presented in the results sections.

### Effects of Arterial Plasticity and Physical Fitness on *a priori* Task-Sensitive ROIs

*A priori* task-sensitive ROIs were defined by the previous publication (Nashiro et al., 2018; Table 2). These ROIs were defined in an independent sample of younger and older adults via whole-brain contrast examining general activation patterns during GSC and LSC while controlling for age. Brain activations from these ROIs were age invariant. Any age-related difference in brain activations found in the current sample therefore could be largely attributed to individual differences in cardiovascular risks among current participants, particularly the older participants.

**TABLE 2:** Task-sensitive *a priori* ROIs defined by Nashiro et al. (2018).

**Cost**	**Contrast**	**H**	**Name**	***X***	***Y***	***Z***	**Cluster Size # Voxels**
GSC	Dual > Single	L	Middle Frontal Gyrus	−30	6	56	248
		R&L	Paracingulate Gyrus	6	14	50	474
		R	Middle Temporal Gyrus	56	−48	−10	607
		R	Middle Frontal Gyrus	46	22	36	1494
		R&L	Supramarginal Gyrus	−42	6	32	14784
LSC	Dual: SW > NS	R	Post-central Gyrus	40	−28	54	241

*ROI, region of interest; GSC, global switch cost; LSC, local switch cost; H, hemisphere; R, right; L, left.*

Mean% signal changes during single and dual blocks were extracted from the five ROIs activated for GSC. Mean% signal changes during switch and non-switch trials were extracted from the one ROI activated for LSC. Repeated measures ANCOVA on mean% signal change with conditions (GSC: single and dual; LSC: switch and non-switch) as within-subject variables and groups (high vs. low arterial plasticity; high vs. low fitness) as between-subject variables were conducted for all cognitive control ROIs, after controlling for age, gender, and BOLD signal variability. Combined effects of arterial plasticity and fitness on cognitive control ROIs were examined by comparing mean% signal changes among the three risk factors groups (2 factors vs. 1 factor vs. 0 factor).

## Results

### Effects of Arterial Plasticity and Physical Fitness on Older Adults Reaction Times During Task

Regression analyses with age, and sex entered as covariate found that MET was significantly predictive of lower GSC reaction time (β = −0.37, *p* = 0.04), suggesting that high fitness in older adults was associated with better dual task maintenance. MET was not predictive of LSC reaction time (β = 0.14, *p* = 0.47). *PsP* was neither predictive of GSC reaction time (β = −0.14, *p* = 0.29), nor predictive of LSC (β = 0.09, *p* = 0.52) in older adults. For behavioral performance in each older adults groups, see Supplementary Table 1.

### Brain Activations Differences Between Older Adults With High Versus Low Arterial Plasticity

**FIGURE 2:**
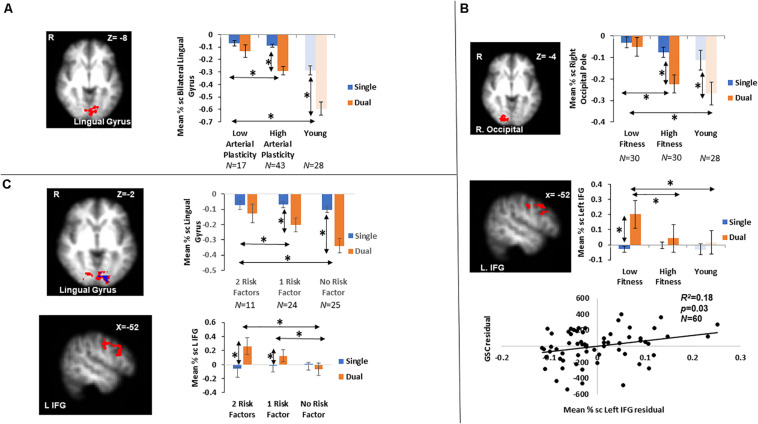
Results from fMRI whole-brain contrasts in older adults. **(A)** Bilateral lingual gyrus identified from the Low > High arterial plasticity contrast in older adults. Younger adults’ data were plotted to show that younger adults were also suppressing this region during GSC. **(B)** The right occipital pole and the left IFG identified from the Low > High physical fitness contrast in older adults. Younger adults’ data were plotted to show that activation patterns in younger adults were similar to those in high fitness older adults. Scatter plot presented residuals from the regression analysis that showed overactivation in left IFG significantly predicted longer GSC reaction time in older adults after controlling for gender, age, and BOLD signal variability. **(C)** Bilateral lingual gyrus (above in red) and left IFG (below) identified from the 0 > 2 risk factors contrast. The small lingual gyrus cluster identified by contrasting 11 older adults with 0 risk factor and 11 older adults with 2 risk factors was presented in blue (above). ^∗^ indicates significant difference at alpha level of 0.05. Error bars represent standard errors of means. Number of participants in each group are noted underneath group name.

A cluster around bilateral lingual gyrus (Table 3) was identified by the contrast between high arterial plasticity (*N* = 43) and low arterial plasticity (*N* = 17) older adults for GSC (Dual mean% signal change -Single mean% signal change). Mean% signal changes from younger controls (*N* = 28) were also extracted to assess age-related differences in this arterial plasticity-sensitive region. Visual inspection of mean% signal changes from this region found that this region was in fact, suppressed by older adults, as well as by younger controls (Figure 2A) during both single and dual tasks. Low arterial plasticity older adults (*N* = 17) were found to have reduced suppression of this region for the GSC contrast (Dual mean% signal change – Single mean% signal change) compared to the younger controls [*t*(44) = 2.9, *p* < 0.01, 95% CI = 0.03 – 0.18]. High arterial plasticity older adults (*N* = 43), however, did not significantly differ in the suppression of this region when compared to the younger controls [*t*(68) = 1.9, *p* = 0.8, 95% CI = −0.09 – 0.07]. Brain-behavior association analysis did not find a significant association between suppressions in this region and task performance in older adults.

**TABLE 3:** Brain regions showing significant activation differences in whole-brain group contrasts.

**Group Contrast**	**Cost Contrast**	***H***	**Name**	***X***	***Y***	***Z***	**Cluster Size # Voxels**	***Z* Max**
**Arterial Plasticity** (Low > High) 17 vs. 43	**GSC** (Dual > Single)	R&L	Lingual Gyrus	−4	−86	−4	429	3.72
**Fitness** (Low > High) 30 vs. 30	**GSC** (Dual > Single)	L	Inferior Frontal Gyrus	−58	18	28	265	3.84
		R	Occipital Pole	24	−86	−6	227	3.67
**Risk Factors** (0 > 2) 11 vs. 25	**GSC** (Dual > Single)	R&L	Lingual Gyrus	−14	−86	−4	822	4
		L	Inferior Frontal Gyrus	−56	22	26	455	3.53

*For each contrast, the numbers of participants included in both groups are listed below the contrast name. For example, in the arterial plasticity group contrast, the number of participants included are: 17 Low arterial plasticity vs. 43 High arterial plasticity. GSC, global switch cost; LSC, local switch cost; H, hemisphere; R, right; L, left; Z Max, Z score from the peak voxel within cluster.*

### Brain Activations Differences Between Older Adults With High Versus Low Physical Fitness

Increased GSC activations were identified in the right occipital pole, and the left inferior frontal gyrus (IFG) in low fitness older adults (*N* = 30) compared to high fitness older adults (*N* = 30; Table 3). For the right occipital pole, visual inspection of mean% signal changes from this region found that this region was suppressed more during dual tasks when compared to single tasks by both high fitness older and younger adults, with low fitness older adults showing nearly no differential suppression between dual and single tasks (Figure 2B). Low fitness older adults had significantly less suppression of this region for the GSC contrast (Dual mean% signal change – Single mean% signal change) compared to the younger controls [*t*(56) = −1.9, *p* = 0.05, 95% CI = 0.004 – 0.16]. High fitness older adults, however, did not significantly differ in suppressing this region for GSC compared to the younger controls [*t*(56) = 1.5, *p* = 0.1, 95% CI = −0.03 – 0.14]. Brain-behavior association analysis did not find a significant association between the differential suppressions in this region and task performance in older adults.

For the left IFG, low fitness older adults were activating this region during dual blocks but not during single blocks (Figure 2B). Given that this region was activated only in older adults, it was a region for overactivation. More specifically, overactivations of left IFG during the dual blocks were significantly higher for low fitness older adults when compared to the younger controls [*t*(56) = −2.45, *p* = 0.02, 95% CI = −0.19 – −0.02]. However, there was no difference in overaction of this region during dual blocks between high fitness older adults and the younger controls [*t*(56) = −1.6, *p* = 0.8, 95% CI = −0.17 – 0.19], suggesting that overactivation in this region during dual tasking could be attributed to low physical fitness in older adults.

Brain-behavior association analysis between GSC RT (Dual RT- Single RT) and mean% signal change of left IFG in the GSC contrast (Dual mean% signal changes – Single mean% signal changes) was conducted on their residuals, after controlling for age, gender, and BOLD signal variability. The results from this regression analysis showed that overactivations in older adults were significantly associated with longer GSC RT (*R*^2^ = 0.18, β = 0.27, *p* = 0.04; Figure 2B), that is, with worse task performance.

### Combined Effects of Arterial Plasticity and Physical Fitness on Older Adults Brain Activations

Contrasting between older adults with 0 risk factor (*N* = 25) and older adults with 2 risk factors (*N* = 11) resulted in two significant clusters in block design analysis (GSC contrast; Table 3 and Figure 2C), a bilateral lingual gyrus cluster that resembled the region defined in section “Brain Activations Differences Between Older Adults With High Versus Low Arterial Plasticity” (Low > High arterial plasticity), and a left IFG cluster that resembled the region defined in section “Brain Activations Differences Between Older Adults With High Versus Low Physical Fitness” (Low > High fitness). Since there were only 11 participants in the 2 risk factors group, a sub-analysis with these 11 high risk older adults and 11 age-matched older adults from the 0 risk factor group was conducted. Whole-brain contrast between 11 older adults with 0 risk factors and older adults with 2 risk factors (*N* = 11) identified a smaller, overlapping cluster of activation within the bilateral lingual gyrus (Figure 2C).

For bilateral lingual gyrus, an ANCOVA with 3 Groups (2 factors vs. 1 factor vs. 0 factor) as the fixed factor, and GSC suppression (Dual mean% signal change – Single mean% signal change) as dependent variable, after controlling for age, gender and BOLD signal variability, found a significant main effect of Group [*F*(2,57) = 4.88, *p* = 0.01, η_*p*_^2^ = 0.15]. Most importantly, a *post hoc* pairwise comparison showed that the 0 risk factor group had significantly larger GSC suppression compared to not only the 2 risk factors group (*M_difference_* = −0.18, *p* < 0.01, 95% CI = −0.33 – −0.04), but also to the 1 risk factor group (*M_difference_* = −0.1, *p* = 0.04, 95% CI = −0.23 – −0.02). There was no significant difference between the 0 risk and 1 risk factor group in GSC suppression (*M_difference_* = 0.08, *p* = 0.14, 95% CI = −0.05 – 0.22). Such result from the 1 risk factor group suggests that the combined effects of low arterial plasticity and low fitness on suppression in the lingual gyrus were greater than the separate effects of arterial plasticity or fitness alone.

For the left IFG, an ANCOVA with dual block activations as the dependent variable and 3 groups (2 factors vs. 1 factor vs. 0 factor) as the fixed factor, after controlling for age, gender and BOLD signal variability, showed a significant main effect of Group [*F*(2,57) = 3.48, *p* = 0.04, η_*p*_^2^ = 0.11]. *Post hoc* pairwise comparison showed that the dual activation in the 1 risk factor health group was significantly different from the 0 risk factor group (*M_difference_* = 0.06, *p* = 0.04, 95% CI = 0.001 – 0.14), but not from the 2 risk factor group (*M_difference_* = −0.02, *p* = 0.6, 95% CI = −0.24 – 0.13). Such result from the 1 risk factor group suggests that the combined effects of low arterial plasticity and low fitness on overactivation in the left IFG was not larger than the separate effects of arterial plasticity or fitness alone.

### Effects of Arterial Plasticity and Physical Fitness on *a priori* Task-Sensitive ROIs

**FIGURE 3:**
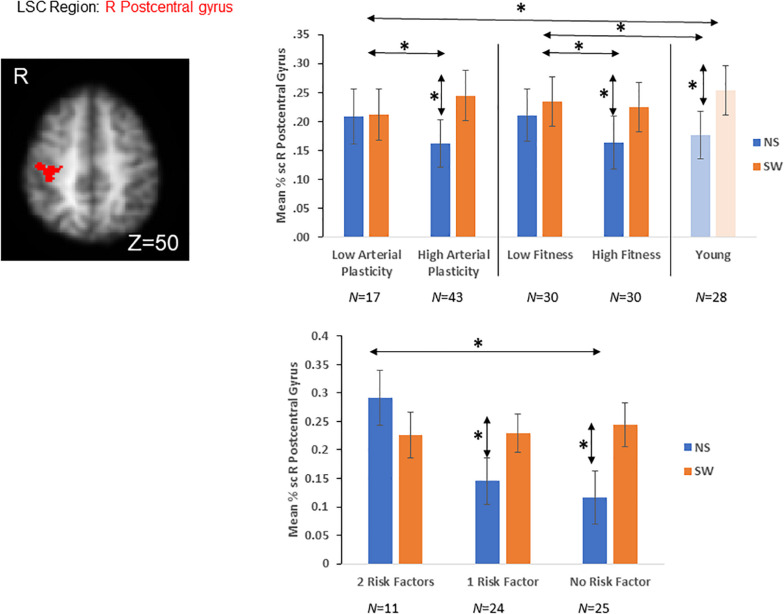
*A priori* LSC ROI defined from literature (Nashiro et al., 2018). Older adults with high arterial plasticity showed significant LSC activations (differences between switch and non-switch trials) in the right post-central gyrus, like that from younger adults. Older adults with low arterial plasticity failed to show LSC activations in this region. When older adults were separated into high and low fitness groups, high fitness older adults also showed increased LSC activations compared to low fitness older adults. In addition, older adults with low arterial plasticity and low fitness (2 risk factors) showed the least LSC activations in this region. *Indicates significant difference at alpha level of 0.05. Error bars represent standard errors of means. NS, Non-Switch trials. SW, Switch trials. Number of participants in each group are noted underneath group name.

In a recent publication (Nashiro et al., 2018), we have identified six task-sensitive ROIs, where activations sensitive to both GSC and LSC were observed (Table 2, Supplementary Figure 1, and Figure 3). Five fronto-parietal ROIs were identified for GSC, and the right post-central gyrus was identified for LSC. Activations from these ROIs were significantly associated with GSC and LSC reaction times in younger adults, suggesting these regions were indeed cognitive control regions needed to successfully perform the task. In addition, activations from these regions did not differ between older and younger adults. In subsequent analyses, we are investigating the effects of arterial plasticity and physical fitness on activations from these task-sensitive ROIs in the current sample of older and younger adults. In particular, if significant age-related difference was observed in the current analyses, such differences could then be largely attributed to cardiovascular risks in the older adults sample.

#### Effects of Arterial Plasticity on *a priori* Task-Sensitive ROIs

A repeated measures ANOVA was conducted, with Plasticity (high vs. low arterial plasticity; 43 vs. 17) as a between-subject variable and Condition (single vs. dual) as a within-subject variable, on mean% signal changes averaged across the five GSC regions (Table 2 and Supplementary Figure 1), after controlling for age, gender and BOLD signal variability (see Supplementary Table 2 for results in each ROI separately). We found no significant main effects of Plasticity [*F*(1,57) = 0.17, *p* = 0.68, η_p_^2^ = 0.01], and no significant Plasticity by Condition interaction [*F*(1,57) = 1.83, *p* = 0.18, η_p_^2^ = 0.03].

A repeated measures ANOVA was conducted, with Plasticity (high vs. low arterial plasticity) as a between-subject variable and Condition (switch vs. non-switch trials) as a within-subject variable, on mean% signal changes in the LSC region (right post-central gyrus; Table 2 and Figure 3), after controlling for age, gender and BOLD signal variability. We did not find any significant main effect of Plasticity [*F*(1,57) = 0.6, *p* = 0.43, η_*p*_^2^ = 0.01], but a significant Plasticity by Condition interaction was observed [*F*(1,57) = 8.32, *p* < 0.01, η_*p*_^2^ = 0.11], such that high arterial plasticity older adults showed increased LSC activation differences (switch mean% signal change – non-switch mean% signal change) compared to low arterial plasticity older adults (Figure 3). Mean% signal change between switch and non-switch trials were extracted for the LSC region (right post-central gyrus) from younger controls, to assess age-related differences in LSC activations. An ANOVA with Age-groups (low arterial plasticity, high arterial plasticity, younger controls) as a fixed factor and LSC activation as the dependent variable found a significant effect of Age-group [*F*(2,86) = 4.36, *p* = 0.02, η_*p*_^2^ = 0.09]. *Post hoc* pairwise comparisons with Sidak correction resulted in a significant difference in LSC activation between low arterial plasticity older adults and younger adults (*M_difference_* = −0.13, *p* = 0.02, 95% CI = − 0.25 – −0.02), but not between high arterial plasticity older adults and younger adults (*M_difference_* = 0.01, *p* = 0.8), 95% CI = −0.8 – 0.1).

#### Effects of Physical Fitness on *a priori* Task-Sensitive ROIs

A repeated measures ANCOVA, with Fitness (high vs. low fitness; 30 vs. 30) as a between-subject variable and Conditions (single vs. dual block) as a within-subject variable, on averaged mean% signal changes across the five GSC regions (Supplementary Figure 1), after controlling for age, gender and BOLD signal variability, showed neither any significant main effect of Fitness [*F*(1,57) = 0.01, *p* = 0.92, η_*p*_^2^ = 0.005], nor any significant interaction between Fitness and Condition [*F*(1,57) = 1.64, *p* = 0.2, η_*p*_^2^ = 0.008].

A repeated measures ANOVA was conducted, with Fitness (high vs. low fitness; 30 vs. 30) as a between-subject variable and Condition (switch vs. non-switch trials) as a within-subject variable, on mean% signal changes in the LSC region, after controlling for age, gender, and BOLD signal variability. There was no significant main effect of Fitness [*F*(1,57) = 0.56, *p* = 0.46, η_*p*_^2^ = 0.01] nor was there any significant interaction between Fitness and Condition [*F*(1,57) = 2.55, *p* = 0.12, η_*p*_^2^ = 0.04]. Mean% signal change from switch and non-switch trials of the LSC region (right post-central gyrus) were extracted from the younger controls, to assess age-related differences in LSC activations. An ANOVA with Age-group (low fitness, high fitness, younger controls) as a fixed factor and LSC activation differences as the dependent variable did not find a significant main effect of Age-group [*F*(2,86) = 0.98, *p* = 0.38, η_*p*_^2^ = 0.02; Figure 3].

#### The Combined Effects of Arterial Plasticity and Physical Fitness on the LSC ROI

A repeated ANCOVA with Group (2 factors, 1 factor, and 0 factor) as a between-subject variable and Condition (switch vs. non-switch) as a within-subject variable on mean% signal change from the LSC region, after controlling for age, gender and BOLD signal variability, showed a significant Group by Condition interaction [*F*(2,56) = 3.23, *p* = 0.04; η_*p*_^2^ = 0.1]. *Post hoc* pairwise comparisons with Sidak correction showed that older adults in the 2 factors group showed significantly reduced LSC activation difference in the right post-central gyrus compared to those in the 0 factor group (*M_difference_* = −0.17, *p* = 0.01, 95% CI = −0.30 – −0.04). There was however no significant difference in LSC activation differences between 1 factor older adults and 0 factor older adults (*M_difference_* = −0.08, *p* = 0.17, 95% CI = −0.02 – 0.23), as well as between 1 factor older adults and 2 factors older adults (*M_difference_* = 0.09, *p* = 0.13, 95% CI = −0.03 – 0.19; Figure 3).

### Ancillary Whole-Brain Regression Analyses With Arterial Plasticity and Fitness as Predictors of Brain Activations in Older Adults

Since both *PsP* and MET are continuous variables, additional whole-brain regression analyses were conducted in older adults to examine brain activations directly correlated with these risk factors. Results from whole-brain regression analysis using *PsP* as a predictor of brain activations in older adults are very similar to results from group contrast analysis presented in section “Brain Activations Differences Between Older Adults With High Versus Low Arterial Plasticity” (see Supplementary Material for detail). Using MET as a predictor did not yield significant cluster.

## Discussion

In sum, the current results show that low arterial plasticity and low physical fitness were separately associated with altered brain activation patterns in older adults. In particular, low arterial plasticity and low physical fitness were separately associated with reduced suppression in the lingual gyrus, while low physical fitness was also associated with maladaptive overactivation in the left IFG. Such results support our first hypothesis that high cardiovascular risks would be associated with attempted compensation in older adults in brain regions beyond task-sensitive ROIs. Results from task-sensitive ROIs, specifically from the LSC ROI, support our second hypothesis that older adults with low plasticity, or 2 risk factors, failed to activate this region in response to task demand. In addition, combination of low arterial plasticity and low physical fitness in older adults had larger effects on efficiently suppressing the lingual gyrus during GSC than separate effects of either factor alone. With the younger functional control data, we were also able to show that older adults with either low arterial plasticity, low fitness or two risk factors had exaggerated age-related differences in brain activations across all examined brain regions.

The effects of the two cardiovascular risk factors were observed in reduced suppression of activity in lingual gyrus/occipital pole regions (both low arterial plasticity and low fitness) and in overactivations of the left IFG (low fitness) during GSC. In cognitive control tasks, such as Flanker and Stroop, successful suppression of activity in the lingual gyrus, a visual brain region, has been related with better task performance in older adults (Gazzaley and D’esposito, 2007). However, in the current study the reduced suppression of lingual gyrus activity was not related to task performance in older adults, suggesting that it could be involved in just the visual cue processing (e.g., responding to the changing background color cues while switching during the dual blocks), but not in cognitive control (e.g., GSC).

The left IFG has been suggested by past meta-analyses as an important region for conflict resolution in younger adults during inhibition tasks, such as the Flanker task (Nee et al., 2007), as well as an important region for inner speech (verbalization) process (Liakakis et al., 2011). It is possible that the current task elicited additional cognitive demands, such as conflict resolution and the need to verbalize task conditions, in low fitness older adults. Therefore, overactivation in the left IFG represented “attempted” compensation in low fitness older adults (Cabeza and Dennis, 2012). The current results suggest that impairments in task-switching in older adults with high cardiovascular risks may need to use additional neural strategy to compensate for tanking performance. Nevertheless, such attempted compensation strategy was not effective in boosting performance, evidenced by negative correlation between IFG overactivation and task performance in older adults.

Furthermore, brain activations associated with cardiovascular risks in anterior and posterior regions observed in the current study coincide with the posterior-anterior shifts in aging (PASA)—reduced activities in posterior, sensory processing, brain regions alongside increases in activities in anterior, cognitive control, brain regions in older adults (Davis et al., 2008). It has been suggested that the anterior brain regions were recruited to compensate for functional degradation in the posterior brain regions (Reuter-Lorenz and Cappell, 2008; Reuter-Lorenz and Park, 2014). Since posterior brain regions are important for sensory input, PASA also suggests an increased demand for top-down regulation to regulate sensory inputs older adults (Pfeifer et al., 2019). Although in the current study the reduced suppression in lingual gyrus was not correlated with cognitive control performance, the overactivation in the left IFG was significantly correlated with GSC reaction time in older adults. The maladaptive overactivation in a cognitive control frontal brain region (e.g., IFG), coupled with reduced suppression of visual processing region (e.g., lingual gyrus), therefore, suggests that cardiovascular risks among older adults might contribute to PASA, especially to the increased anterior brain region activation.

Combined effects of the two cardiovascular risk factors were observed in two brain clusters, one resembling an arterial plasticity-sensitive region (bilateral lingual gyrus) and another resembling a fitness-sensitive region (left IFG). The effects of having two risk factors was greater than having one factor alone in the bilateral lingual gyrus. The greater combined effects of 2 risk factors than 1 risk factor on activations of the bilateral lingual gyrus could be due to the underlying blood supply to the region. A postmortem dissection study reported more arterial wall thickening and less elasticity in posterior brain arteries than in anterior brain arteries in older adults (Roth et al., 2017). Recent imaging studies have reported altered regional cerebral blood flow, especially in posterior brain regions, in healthy older adults (Jefferson et al., 2018; Suri et al., 2019). Such pathological differences in blood vessels and cerebral blood flow could result in differential fMRI BOLD signals between anterior and posterior brain regions (Kastrup et al., 1999). Although we have employed MELODIC cleaning of vascular artifacts from BOLD signals and have controlled for BOLD signal variability, it was still possible that posterior regions were more sensitive to differences in cardiovascular risks than anterior regions. However, future studies should examine effects of different cardiovascular risk factors on more brain regions to test such possibility.

In addition to examining effects of cardiovascular risk factors on older adults’ brain activation patterns, the current study investigated effects of risk factors on age-related differences in brain activations during task-switching. From our previous publication, age-invariant, task-sensitive brain activation were defined for cognitive controls (GSC and LSC) in multiple ROIs using the same paradigm (Basak et al., 2018; Nashiro et al., 2018). The current results show that in the LSC ROI (right post-central gyrus) where younger adults and low risk older adults had significant LSC activation difference (between switch and non-switch activations), older adults with high cardiovascular risk failed to show any LSC activation difference. Such results show that in the right post-central gyrus, where age-related difference in cognitive control (LSC) activation was absent in previous publication, high cardiovascular risk in older adults in the current sample led to significant age-related activation difference. Similar results were also observed in arterial plasticity and fitness sensitive regions (i.e., lingual gyrus and left IFG), where older adults with high cardiovascular risk (low arterial plasticity or low fitness) consistently showed larger difference in activation (or suppression) than low risk older adults when compared to younger controls. The current results suggest that some age-related differences in brain activations during task-switching reported by past neuroimaging studies could be potentially attributed to individual differences in cardiovascular risks among older participants.

For the task-sensitive LSC ROI, when older adults were separated based on the number of risk factors they carry, results showed that only older adults with 2 risk factors were not able to show any LSC activation difference. This result, together with results from whole-brain analysis examining combined effects of the two risk factors in older adults, emphasized the importance of maintaining good cardiovascular health, and in turn healthy brain functioning in anterior brain regions, in older adults.

For the current study, a proxy measure of physical fitness was used instead of subjecting frail older adults to an exercise test. Such approach allowed us to collect cardiovascular measures on a large number of older adults, some of whom may refuse or be unable to complete an exercise test to obtain individual VO_2_Max. With this proxy measure of VO_2_Max the current study was able to show significant differences in brain activations between older adults with high and low physical fitness. Future studies could therefore consider applying such easily accessible data collection methods to avoid potential selection bias in older participants.

Even with the simple methods, the current study still had a relatively small sample of high risk older adults, thus resulting in unequal groups when categorizing older adults into different sub-groups. In particular, there were only 11 participants with 2 risk factors. An additional whole-brain analysis contrasting these 11 participants with age-matched participants from the 0-risk factor group found a small, but importantly, overlapping cluster with the lingual gyrus region, suggesting the robustness of the results even in small samples. Related to small sample size of high risk older adults, in older adults with 1 risk factor only there were only six older adults with low arterial plasticity and high fitness. Such small number made it impossible to directly compare the separate effects of arterial plasticity and fitness within the 1 risk factor group thereby assessing which of the two factors are more important in affecting brain activations in older adults. Although we utilized targeted recruitment strategies to reach more high risk older adults, many of them could not participate in the current study because they are ineligible for MRI scans (e.g., using pacemaker). The current, relatively healthy, sample of older adults might have contributed to the limited brain regions with significant effects of cardiovascular risks on activations. However, our were still able to identify brain region where activations were sensitive to the limited range of cardiovascular risk in the current sample of older adults. It is likely that effects of cardiovascular risk factors on older adults with severe hypertension or on obese older adults would be greatly exaggerated compared to those reported by the current study.

## Conclusion

In sum, the current study showed significant effects of two important cardiovascular risk factors, arterial plasticity and physical fitness, on brain activations during task-switching in older adults using easily accessible measures. Older adults with high cardiovascular risks showed maladaptive overactivation in a frontal cognitive control region. Such anterior brain region overactivation might contribute to the PASA model of aging brains. The combined effects of two risk factors were greater than the effects of either factor alone on suppression of posterior visual regions. Most importantly, older adults with high cardiovascular risk showed exaggerated age-related differences in brain activations than those with low cardiovascular risk. Such finding is critical for future aging neuroimage researchers to consider when recruiting older participants, as individual differences in cardiovascular risks among older participants could potentially bias age-related differences in brain activations.

## Data Availability Statement

The raw data supporting the conclusions of this article will be made available by the authors, without undue reservation.

## Ethics Statement

The study received IRB approval from University of Texas South Western Medical Center IRB board as well. Participants signed consent forms from both UT Dallas and UT south western.

## Author Contributions

SQ analyzed the data, interpreted the data, and drafted the manuscript for intellectual content. CB designed and conceptualized the study, revised the manuscript for intellectual content. Both authors contributed to the article and approved the submitted version.

## Conflict of Interest

The authors declare that the research was conducted in the absence of any commercial or financial relationships that could be construed as a potential conflict of interest.
