# Supplementary material for: Influence of Multiple Cardiovascular Risk Factors on Task-Switching in Older Adults: An fMRI Study
Source: Front Hum Neurosci. 2020 Sep 9;14:561877. doi: 10.3389/fnhum.2020.561877 (PMC7509111; doi:10.3389/fnhum.2020.561877)
Supplement: Supplementary file 1 [file Table_1.DOCX]

**Supplementary Material**

| **Supplementary Table 1.** Mean and Standard Deviation for Accuracy, GSC, and LSC from the Four Older Adults Groups | | | | |
| --- | --- | --- | --- | --- |
|  | Older Adults Groups | | | |
| Behavioral Measure | High Arterial Plasticity  *N*=43 | Low Arterial Plasticity  *N*=17 | High Fitness  *N*=30 | Low Fitness  *N*=30 |
|  | M (SD) | M (SD) | M (SD) | M (SD) |
| Overall ACC | 0.93 (0.06) | 0.93 (0.1) | 0.93 (0.09) | 0.93 (0.08) |
| GSC RT | 557.27 (253.96) | 604.33 (172.13) | 571.79 (166.41) | 604.88 (229.57) |
| LSC RT | 124.71 (124.39) | 115.79 (111.49) | 127.43 (125.14) | 120.53 (107.65) |
| ***Note.*** ACC = accuracy; RT = response time; ns = non-significant; GSC = global task-switch cost; LSC = local task-switch cost. | | | | |

**Behavioral performance of older adults**

**The effects of arterial plasticity on activations in the GSC ROIs**

Repeated measures ANCOVAs, with Plasticity (high vs. low) as a between-subject variable and Conditions (single vs dual block) as a within-subject variable controlling for age, gender and BOLD signal variability, on mean % signal changes in each of the five GSC regions (Table 2, Supplementary Figure 1) were conducted. Results from these five repeated ANOCVAs are summarized in Supplementary Table 2.

**The effects of physical fitness on activations in the GSC ROIs**

Repeated measures ANCOVAs, with Fitness (high vs. low fit) as a between-subject variable and Conditions (single vs dual block) as a within-subject variable controlling for age, gender and BOLD signal variability, on mean % signal changes in each of the five GSC regions (Table 2, Supplementary Figure 1) were conducted. Results from these five repeated ANOCVAs are summarized in Supplementary Table 2.


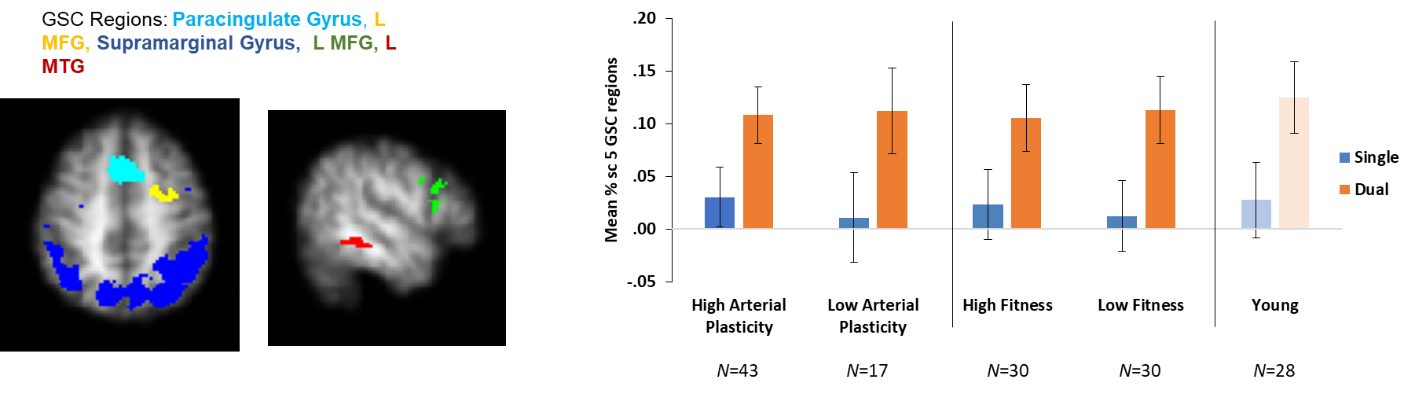


Supplementary Figure 1. No difference in brain activations patterns between: high and low plasticity older adults, high and low fit older adults, and older and younger adults.

| **Supplementary Table 2.** Repeated ANCOVA results for the effects of arterial plasticity on brain activations in the five GSC ROIs in older adults | | |
| --- | --- | --- |
| ROIs | *F* (1, 57) | *p* |
| **Left Middle Frontal Gyrus** |  |  |
| Main effect of Plasticity | 1.75 | 0.2 |
| Condition by Plasticity interaction | 0.33 | 0.59 |
| **Bilateral Paracingulate Gyrus** |  |  |
| Main effect of Plasticity | 0.32 | 0.57 |
| Condition by Plasticity interaction | 0.38 | 0.54 |
| **Right Middle Temporal Gyrus** |  |  |
| Main effect of Plasticity | 0.01 | 0.92 |
| Condition by Plasticity interaction | 0.37 | 0.55 |
| **Right Middle Frontal Gyrus** |  |  |
| Main effect of Plasticity | 0.77 | 0.38 |
| Condition by Plasticity interaction | 0.03 | 0.87 |
| **Bilateral Supramarginal Gyrus** |  |  |
| Main effect of Plasticity | 1.17 | 0.28 |
| Condition by Plasticity interaction | 0.46 | 0.49 |

**Whole-brain regression analysis with *PsP* and MET as continuous predictors of brain activations in older adults**

Regression analysis for the GSC contrast (Dual mean % signal change – Single mean % signal change) identified two posterior brain clusters, where activations were associated with increasing *PsP*. These two regions were the right occipital pole and the left lateral occipital cortex (Supplementary Figure 2). Mean % signal changes from younger controls were also extracted to assess age-related differences in these risk-sensitive regions. Visual inspection of the GSC contrast (that is, the difference in mean % signal changes of Single from Dual) from these two regions found that the positive associations between activations and *PsP* were driven by reduced suppression of these two regions in low arterial plasticity older adults, compared to high arterial plasticity older adults. Compared to the younger controls, low arterial plasticity older adults had reduced suppressions of these regions for the GSC contrast [right occipital pole: *t* (44) =2.8, *p*<0.01; left lateral occipital cortex: *t* (44) =2.9, *p*<0.01]. High arterial plasticity adults, however, did not significantly differ in suppressions of these regions compared to younger controls during GSC contrast [right occipital pole: *t* (68) =1.3, *p*=0.81; left lateral occipital cortex: *t* (44) =1.9, *p*=0.78].

**
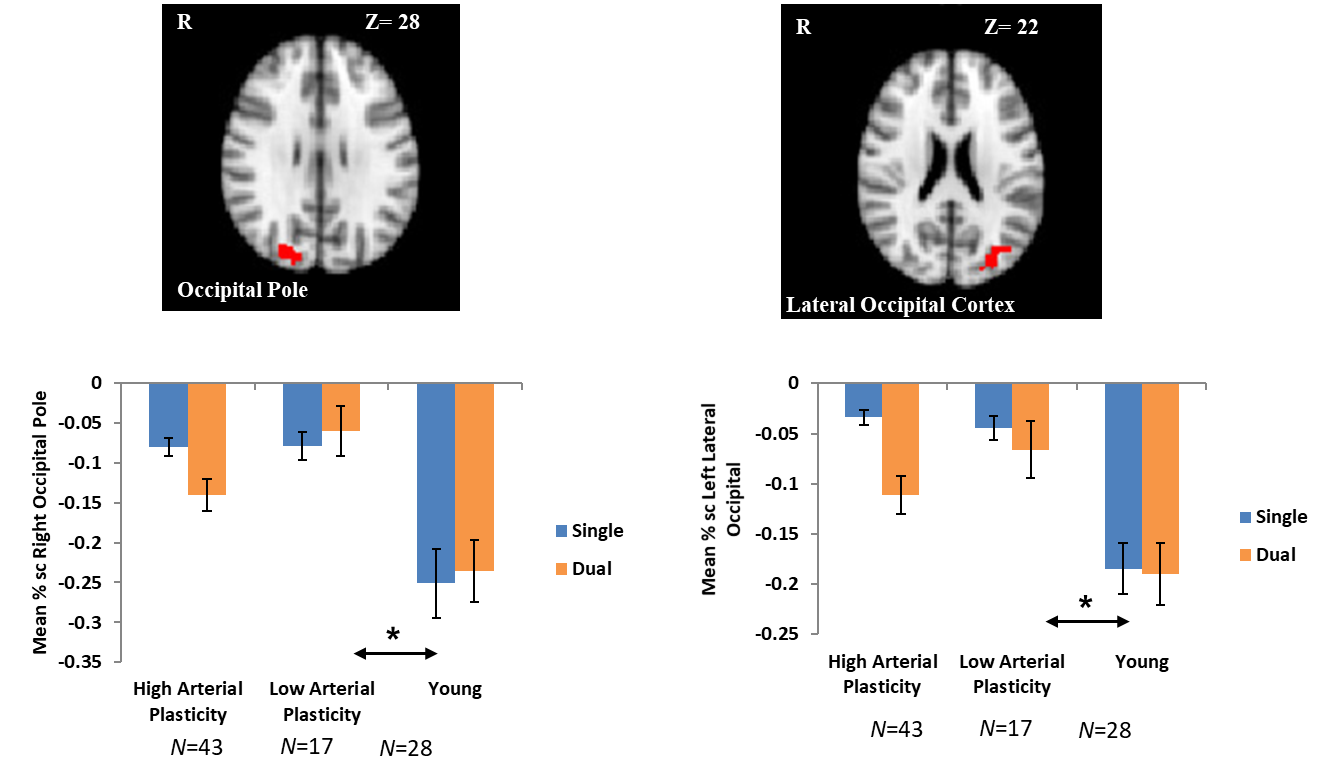
**

**Supplementary Figure 2.** Right occipital pole and left lateral occipital cortex, from whole-brain regression with *PsP* as a predictor, had reduced suppression in low arterial plasticity older adults compared to high arterial plasticity older adults for GSC (Dual *mean % signal changes* – Single *mean % signal changes*). Low arterial plasticity older adults showed significantly reduced GSC neural modulation of this region compared to younger adults, indicated by *. Error bars are standard errors of the mean.

Whole-brain regression analysis with MET as a continuous predictor did not result in any significant cluster.
